# Enantioselective Solvent-Free Synthesis of 3-Alkyl-3-hydroxy-2-oxoindoles Catalyzed by Binam-Prolinamides

**DOI:** 10.3390/molecules200712901

**Published:** 2015-07-16

**Authors:** Abraham Bañón-Caballero, Jesús Flores-Ferrándiz, Gabriela Guillena, Carmen Nájera

**Affiliations:** Departamento de Química Orgánica, Instituto de Síntesis Orgánica, Universidad de Alicante, Apdo. 99, Alicante E-03080, Spain; E-Mails: abraham.banon@ua.es (A.B.-C.); jflores@ua.es (J.F.-F.); cnajera@ua.es (C.N.)

**Keywords:** isatin, aldol, enantioselectivity, solvent-free, prolinamide, supported catalyst

## Abstract

BINAM-prolinamides are very efficient catalyst for the synthesis of non-protected and *N*-benzyl isatin derivatives by using an aldol reaction between ketones and isatins under solvent-free conditions. The results in terms of diastereo- and enantioselectivities are good, up to 99% *de* and 97% *ee*, and higher to those previously reported in the literature under similar reaction conditions. A high variation of the results is observed depending on the structure of the isatin and the ketone used in the process. While 90% of *ee* and 97% *ee*, respectively, is obtained by using (*R*_a_)-BINAM-l-(bis)prolinamide as catalyst in the addition of cyclohexanone and α-methoxyacetone to free isatin, 90% *ee* is achieved for the reaction between *N*-benzyl isatin and acetone using *N*-tosyl BINAM-l-prolinamide as catalyst. This reaction is also carried out using a silica BINAM-l-prolinamide supported catalyst under solvent-free conditions, which can be reused up to five times giving similar results.

## 1. Introduction

**Figure 1:**
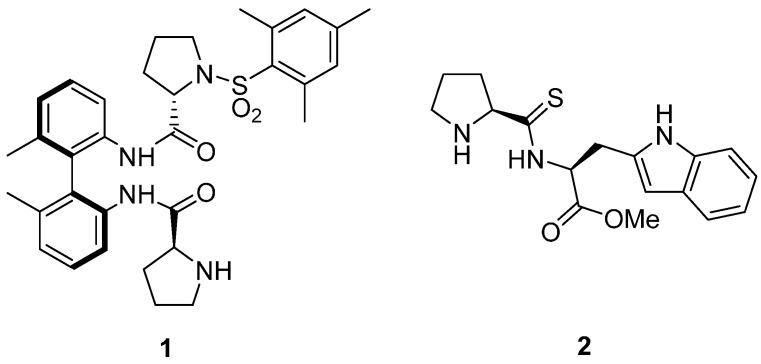
Prolinamides used in the solvent-free conditions aldol reaction of isatins.

Isatin (1*H*-indole-2,3-dione) is a natural product found in different plants, as well as in the parotid gland secretions of Bufo frogs and also in humans metabolism as an endogenous compound [1,2]. This molecule and its derivatives have attracted the interest of the scientific community due to their relevant properties as drug candidates and interesting biological activities [3,4,5]. Chiral 3-substituted-3-hydroxyindolin-2-ones, having a quaternary stereocenter, are found as a core structures in several natural products and pharmaceutically active compounds [6], with its biological activity being determined by the nature of the alkyl group at the C3 position and its absolute configuration. The synthesis of this type of chiral derivatives can be efficiently accomplished by an enantioselective organocatalyzed reaction of free or *N*-protected isatin with enolizable compounds [7]. Following this strategy, the application of the organocatalyzed aldol reaction [8,9,10,11,12,13] has played a major role in the synthesis of isatin derivatives. The use of proline [14,15] and its derivatives [16,17,18,19,20,21,22,23,24] has been described for this transformation achieving typically high yields and enantioselectivities ranging from 75% to 95% *ee*. In most cases, the nucleophilic ketone acted as nucleophile and also as solvent. However, recently the use of two prolinamides derivatives (**1** and **2**) have been reported to promote this aldol reaction under solvent free conditions [25] (Figure 1). While prolinamide **1** (5 mol %) [26] was used in combination with TFA (7.5 mol %) in the addition of 2 equiv. of acetone or cyclohexanone to free isatin at 0 °C using conventional stirring, thiodipeptide **2** (10 mol %) [27] required the use of 3 equiv. of nucleophiles and ball-mill stirring (2760 rpm) at −20 °C, both affording the corresponding aldol products in moderate enantioselectivities (53%–86% *ee*).

**Figure 2:**
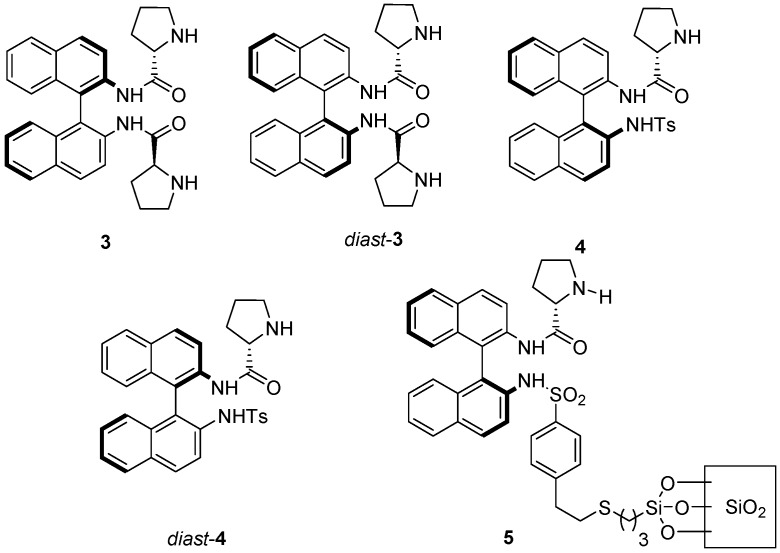
BINAM-prolinamides used under solvent-free conditions for the aldol reaction.

On the other hand, different 1,1′-binaphthyl-2,2′-diamine (BINAM) prolinamide derivatives (Figure 2) has been tested as catalysts for the intra- and intermolecular aldol reaction between cyclic, acyclic, alkyl and α-functionalized ketones and aldehydes under different reaction conditions [28,29,30,31,32,33,34,35,36,37,38,39,40,41], including solvent-free conditions. Thus, BINAM-l-(bis)prolinamide 3 [42,43,44] and *N*-tosyl BINAM-l-prolinamide derivative **4** [45,46,47,48,49,50] and even their supported analogues [51,52,53,54,55], such as the silica derivative **5**, have shown to be very effective catalysts for the aldol reaction. Therefore, we decided to evaluate the use of these binam-prolinamide derivatives in the aldol reaction of ketones with isatins under solvent-free conditions.

## 2. Results and Discussion

**Scheme 1:**
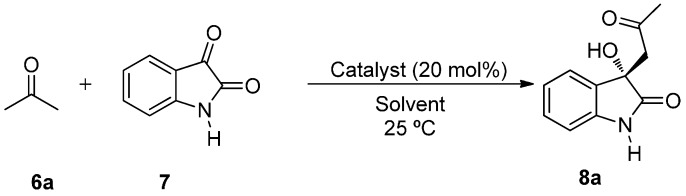
BINAM-prolinamide catalyzed reaction between acetone and isatin.

The aldol reaction between acetone **6a** (3 equiv.) and non-protected isatin **7** with a catalyst loading of 20 mol % at room temperature was chosen as benchmark to test the efficiency of catalyst **3** under different reaction media (Scheme 1).

The reaction proceeded very slowly using different type of polar and apolar solvents and even in water (entries 1–6, Table 1), with low conversions and enantioselectivities being achieved. However, when the process was carried out under solvent-free conditions, the reaction rate increased tremendously, achieving the expected product **8a** after only 6h with almost full conversion, but unfortunately in low *ee* (entry 7, Table 1). In order to improve the enantioselectivity, the reaction temperature was decreased to 0, −20, −50 and −70 °C (entries 8–11, Table 1), just an increase of the reaction time was observed without enantioselectivity enhancement. We have observed in previous aldol processes that the addition of a small amount of benzoic acid [30] and water provoked an increase on the reaction rate. Therefore, the use of these two additives was evaluated (entries 12–14, Table 1), but again a deleterious effect on the enantioselectivities was observed. The effect of the decrease in the amount of catalyst was checked (entries 15–16, Table 1), affording with only a 5 mol % of catalyst, product **8a** in similar results than those obtained by using 20 mol %, with more time being required for the reaction completion. The reduction of the amount of the used ketone to 2 or 1 equiv. gave similar results in terms of enantioselectivities, but longer reaction times and lower conversion was found when only 1 equiv. of ketone (entries 17–18, Table 1) was used. The use of acetone as reaction media didn’t led to any improvement of the result (entry 19, Table 1). Thus, using only 2 equiv. of acetone and performing the reaction at room temperature and just 5 mol % of catalyst **3**, the performance of the diastereoisomer of compound **3**, (*S*_a_)-binam-l-prolinamide (*diast*-**3**), *N*-tosyl-(*S*_a_)-binam-l-prolinamide **4** and its diastereoisomer *N*-tosyl-(*R*_a_)-binam-l-prolinamide (*diast*-**4**) was studied. While (*S*_a_)-binam-l-prolinamide (*diast*-**3**) gave product **8a** with similar results to those encountered with catalyst **3** (entry 20, Table 1), *N*-tosyl-(*S*_a_)-binam-l-prolinamide **4** and its diastereoisomer led to lower conversions in longer reaction times (entries 21–22, Table 1).

**Table 1:** Optimization of reaction conditions.

Entry	Solvent ^a^	Cat. (mol %)	Ketone (equiv.)	T ^a^ (°C)	*t*	Conv (%) ^b^	*ee* (%) ^c^
1	DMSO	3(20)	3	25	3 days	<50	12
2	THF	3 (20)	3	25	3 days	<30	21
3	Hexane	3 (20)	3	25	3 days	<20	14
4	H_2_O:DMF	3 (20)	3	25	3 days	<20	0
5	DMF	3 (20)	3	25	3 days	<20	0
6	H_2_O	3 (20)	3	25	3 days	<30	11
7	–	3 (20)	3	25	6 h	>99	29
8	–	3 (20)	3	0	1 days	83	27
9	–	3 (20)	3	−20	3 days	74	31
10	–	3 (20)	3	−50	3 days	<50	33
11	–	3 (20)	3	−70	3 days	<30	30
12 ^d^	–	3 (20)	3	25	6 h	>99	22
13 ^d,e^	–	3 (20)	3	25	4 h	>99	17
14 ^e^	–	3 (20)	3	25	4 h	>99	20
15	–	3 (5)	3	25	1 days	>99	31
16	–	3 (10)	3	25	1 days	>99	28
17	–	3 (5)	2	25	1 days	>99	30
18	–	3 (5)	1	25	1 days	82	27
19	Acetone	3 (5)	excess	25	1 days	>99	22
20	–	*diast-*3 (5)	2	25	1 days	>99	20
21	–	4 (5)	2	25	3 days	<30	24
22	–	*diast-*4 (5)	2	25	3 days	<30	29

^a^: 0.15 mL for 0.3 mmol of **7** was used; ^b^: Conversion based on the amount of the unreacted isatin; ^c^: Determined by chiral phase HPLC analysis; ^d^: 5 mol % PhCO_2_H was used; ^e^: 10 equiv. of water was added.

**Scheme 2:**
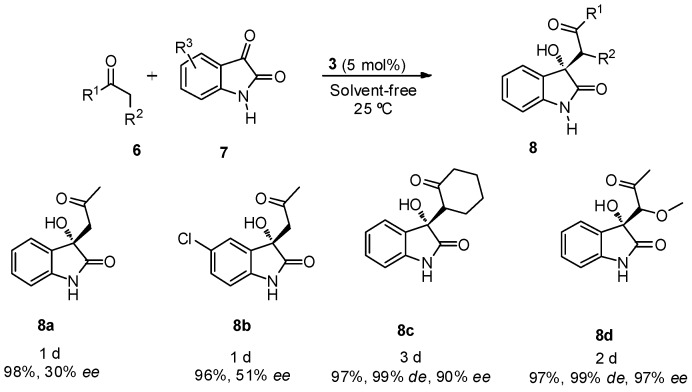
BINAM-prolinamide **3** catalyzed reaction between ketone and free isatins.

The best reaction conditions, 5 mol % of catalyst **3** at room temperature and under solvent-free reaction conditions were applied in the reaction between different ketones **6** (2 equiv.) and free isatins **7** (Scheme 2). Good yields were achieved for all the assayed ketones, with good diastereo- and enantioselectivities when cyclohexanone and α-methoxyacetone were used as nucleophiles, although a longer reaction time was required for the reaction completion. Product **8c** was obtained mainly as its *anti*-isomer in high diastereoselectivity.

When α-methoxyacetone was used as nucleophile, the reaction took place through the methylene group, being the main diastereoisomer achieved the *anti-*product **8d**. The same regioselectivity was observed in the reaction with other ketones, such as butanone, α-methylsulfanylacetone or α-chloroacetone, but unfortunately these products were achieved as racemic mixtures. Also, attempts to use aldehydes, as nucleophiles, for this kind of transformation led to the formation of racemic mixtures.

**Scheme 3:**
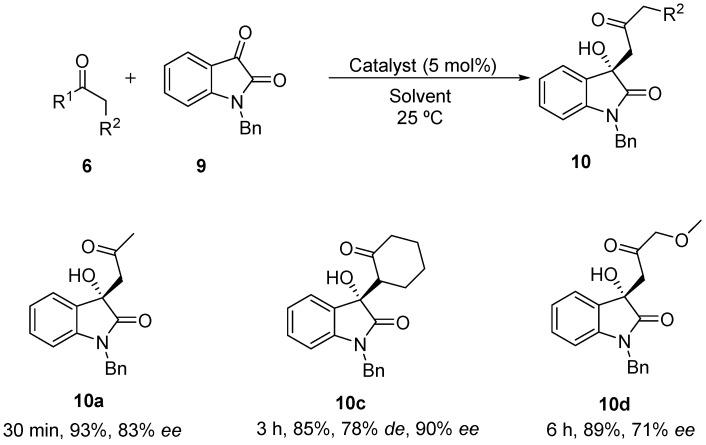
Solvent-free BINAM-prolinamide catalyzed reaction between acetone and *N*-benzyl isatin.

As compounds **8** and free isatins **7** are rather insoluble in most solvents, a more soluble *N*-benzyl isatin **9** was tested in the aldol reaction with acetone (Scheme 3).

First, the performance of the different catalysts under solvent-free conditions was evaluated, using just 5 mol % of catalyst loading (Table 2). As expected, the higher solubility of the electrophile decreases the heterogeneousity of the reaction, with the expected product being formed in only minutes. Surprisingly, catalyst **3** and its diastereoisomer led to the racemic product in the reaction with acetone (entries 1 and 2, Table 2), while catalyst **4** led to product **10** with up to 83% *ee* (entry 3, Table 2). When *N*-tosyl-(*R*_a_)-binam-l-prolinamide (*diast*-**4**) was used as catalyst, product **10** was obtained with lower enantioselectivity (entry 4, Table 2), showing that in catalyst **4** as synergistic effect between the axial chirality of the binaphthyl framework and the proline takes place. Attempts to improve the catalytic efficiency of **4** by adding water, benzoic acid or both failed (entries 5–7, Table 2). When cyclohexanone was used as nucleophile in this reaction, only catalyst **3** showed to be active. Using this catalyst, product **10c** was achieved as *anti*-isomer, but in lower diastereoselectivity compared to the one achieved in the reaction with the free isatin **7**. Also, an unexpected result was achieved when α-methoxyacetone was used as nucleophile with a reverse regioselectivity being found. The regioisomer **10d** was the only product formed in a 71% *ee*.

**Figure 3:**
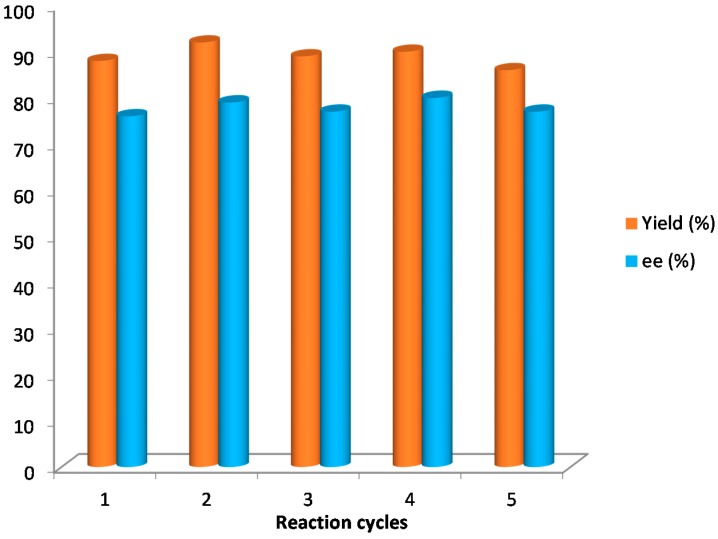
Recycling study of catalyst **5** in the synthesis of **10a**.

Finally, the silica-supported catalyst **5** (10 mol %) was applied under solvent-free conditions for the synthesis of compound **10a**, its recovery and recyclability being studied. The results achieved were similar in terms of yields and enantioselectivities to those achieved by using catalyst **4**. The catalyst was recovered by filtration after reaction completion and reused five times without observing any changes in the results (Figure 3).

**Table 2:** Optimization of reaction conditions.

Entry	R1,R2	Cat. (mol %)	Product	Ketone (equiv.)	T ^a^ (°C)	*t* (min)	Conv (%) ^a,b^	*de* (%) ^c^	*ee* (%) ^d^
1	Me,H	**3**	**10a**	2	25	5	>99	-	0
2	Me,H	*diast*-**3**	**10a**	2	25	5	>99	-	0
3	Me,H	**4**	**10a**	2	25	30	>99 (93)	-	83
4	Me,H	*diast*-**4**	**10a**	2	25	90	>99	-	75
5 ^e^	Me,H	**4**	**10a**	2	25	30	>99	-	70
6 ^f^	Me,H	**4**	**10a**	2	25	30	>99	-	59
7 ^e,f^	Me,H	**4**	**10a**	2	25	30	>99	-	63
8	(CH_2_)_4_	**3**	**10c**	2	25	90	90 (85)	78	90
9	Me,OMe	**3**	**10d**	2	25	360	92 (89)	-	71

^a^: Conversion based on the amount of the unreacted isatin; ^b^: In parenthesis, yield after column chromatography purification; ^c^: Determined by ^1^H-NMR; ^d^: Determined by chiral phase HPLC analysis; ^e^: 5 mol % PhCO_2_H was used; ^f^: 10 equiv. of water was added.

## 3. Experimental Section

### 3.1. General Information

All reactions for catalyst preparation were carried out under argon. Dry DMF, dry toluene, dry DCM, dry THF, pyridine, and triethylamine and all others reagents were commercially available and used without further purification. ^1^H-NMR (300 or 400 MHz) and ^13^C-NMR (75 or 100 MHz) spectra were obtained at 25 °C with CDCl_3_ as solvent and TMS as internal standard, unless otherwise stated. HPLC analyses were performed with a chiral column (detailed for each compound below), with mixtures of *n*-hexane/isopropyl alcohol as mobile phase, at 25 °C. Analytical thin-layer chromatography (TLC) was performed on silica gel plates and the spots were visualized under UV light (λ = 254 nm). For flash chromatography silica gel 60 (0.063–0.2 mm) was employed.

Catalysts **3**, *diast*-**3**, **4** and *diast-***4** were prepared as previously described [43,48].

### 3.2. Procedure for the Addition of Ketones to Isatins

To the mixture of the corresponding isatin (0.3 mmol), and catalyst **3** or **4** (5 mol %), ketone (0.6 mmol) was added. The reaction was stirred using magnetic stirring until the isatin was consumed (monitored by TLC), then the crude product was purified by a silica gel chromatography.

### 3.3. Procedure for Catalysis Recover and Reuse

After each cycle, the reaction was quenched by filtration and the resin was washed with a 0.5 M solution of NaOH, to remove the benzoic acid occluded in the polymeric matrix. Then, the resin was washed several times either with ethyl acetate, ethanol or acetone and dried under vacuum. The same resin was used in the following reaction cycle.

### 3.4. Physical, Analytical and Spectal Data

*(R)-3-Hydroxy-3-(2-oxopropyl)indolin-2-one* (**8a**) [56]. Yield 98%; ^1^H-NMR (400 MHz, CDCl_3_,): δ 7.63 (d, *J* = 7.8 Hz, 1H), 7.37 (d, *J* = 7.6 Hz, 1H), 7.07 (t, *J* = 7.4 Hz, 1H), 6.89 (d, *J* = 7.6 Hz, 1H), 3.20 (d, *J* = 17.2 Hz, 1H), 2.98 (d, *J* = 17.2 Hz, 1H), 2.22 (s, 3H); ^13^C-NMR (100 MHz, CDCl_3_) δ 207.4, 179.8, 137.0, 132.3, 128.2, 125.3, 123.0, 112.7, 76.2, 49.5, 31.6, 49.5, 76.2, 112.7. HPLC Chiralcel OJ column, *n*-hexane/2-propanol = 80:20, flow rate 0.8 mL/min, λ = 254 nm; t_R_ 15.34 min (major) and 19.22 min (minor).

*(S)-5-Chloro-3-hydroxy-3-(2-oxopropyl)indolin-2-one* (**8b**) [56]. Yield 96%; ^1^H-NMR (300 MHz, MeOD) δ 7.37–7.29 (m, 1H), 7.24 (dd, J = 8.3, 2.2 Hz, 1H), 6.91–6.80 (m, 1H), 3.41 (d, *J* = 17.2 Hz, 1H), 3.20 (d, *J* = 17.3 Hz, 1H), 2.08 (s, 3H). ^13^C-NMR (75 MHz, MeOD) δ 207.2, 180.7, 142.4, 134.3, 130.4, 128.5, 125.2, 112.3, 74.6, 50.9, 30.4. HPLC Chiralcel OJ column, *n*-hexane/2-propanol = 80:20, flow rate 0.8 mL/min, λ = 254 nm; t_R_ 23.12 min (major) and 35.44 min (minor).

*(S)-3-Hydroxy-3-((R)-2-oxocyclohexyl)indolin-2-one* (**8c**). Yield 97%; ^1^H-NMR (400 MHz, DMSO) δ 10.18 (s, 1H), 7.17 (ddd, *J* = 11.2, 8.8, 4.3 Hz, 2H), 6.83 (ddd, *J* = 22.7, 14.6, 4.3 Hz, 2H), 5.80 (s, 1H), 3.07 (dd, *J* = 13.1, 5.2 Hz, 1H), 2.59 (ddd, *J* = 12.4, 5.1, 2.6 Hz, 1H), 2.40–2.25 (m, 1H), 2.10–1.99 (m, 1H), 1.94 (dt, *J* = 22.7, 8.8 Hz, 2H), 1.86–1.76 (m, 1H), 1.67 (dt, *J* = 12.6, 3.2 Hz, 1H), 1.55–1.37 (m, 1H). ^13^C-NMR (101 MHz, DMSO) δ 209.1, 178.6, 143.4, 130.8, 128.5, 126.3, 124.7, 120.8, 109.3, 73.8, 57.3, 41.4, 26.6, 24.4. Elemental analysis C_14_H_15_NO_3_: C 68.56, H 6.16, N 5.71. Found C 69.19, H 6.32, N 5.65. HPLC Chiralpak AS-H column, *n*-hexane/2-propanol = 70:30, flow rate 1.0 mL/min, λ = 254 nm; t_R_ 27.34 min (major) and 43.12 min (minor).

*(R)-3-Hydroxy-3-((R)-1-methoxy-2-oxopropyl)indolin-2-one* (**8d**). Yield 85%; ^1^H-NMR (300 MHz, MeOD) δ 7.53–7.46 (m, 1H), 7.25 (td, *J* = 7.7, 1.3 Hz, 1H), 7.02 (td, *J* = 7.6, 0.9 Hz, 1H), 6.83 (d, *J* = 7.7 Hz, 1H), 4.03 (s, 1H), 3.54 (s, 3H), 1.99 (s, 3H). ^13^C-NMR (75 MHz, MeOD) δ 210.5, 179.0, 143.5, 131.0, 130.3, 126.3, 123.3, 111.1, 91.3, 78.6, 61.4, 27.4. Elemental analysis C_12_H_13_NO_4_: C 61.27, H 5.57, N 5.95. Found C 62.02, H 5.69, N 5.87. HPLC Chiralpak AS-H column, *n*-hexane/2-propanol = 80:20, flow rate 1.0 mL/min, λ = 254 nm; t_R_ 34.46 min (major) and 38.23 min (minor).

*(S)-1-Benzyl-3-hydroxy-3-(2-oxopropyl)indolin-2-one* (**10a**) [56]. Yield 97%; ^1^H-NMR (300 MHz, CDCl_3_) δ 7.39–7.22 (m, 13H), 7.19 (td, *J* = 7.8, 1.3 Hz, 2H), 7.02 (td, *J* = 7.6, 1.0 Hz, 2H), 6.69 (d, *J* = 7.8 Hz, 2H), 4.95 (d, *J* = 15.8 Hz, 2H), 4.83 (d, *J* = 15.8 Hz, 2H), 4.54 (s, 2H), 3.27 (d, *J* = 17.0 Hz, 2H), 3.06 (d, *J* = 17.1 Hz, 2H), 2.16 (s, 6H). ^13^C-NMR (75 MHz, CDCl_3_) δ 207.0, 176.4, 142.7, 135.3, 129.7, 128.7, 127.6, 127.1, 123.7, 123.1, 109.6, 77.4, 77.0, 76.5, 74.1, 49.0, 43.8, 31.2. HPLC Chiralpak AD-H column, *n*-hexane/2-propanol = 90:10, flow rate 1.0 mL/min, λ = 280 nm; t_R_ 27.34 min (minor) and 33.12 min (major).

*(R)-1-Benzyl-3-hydroxy-3-((S)-20-oxocyclohexyl)indolin-2-one* (**10c**) (Product CAS number 881081-60-3). Yield 93%; ^1^H-NMR (300 MHz, CDCl_3_): δ 1.54–2.00 (m, 6H) 2.28–2.47 (m, 2H), 2.97–3.06 (m, 1H,) 3.20–3.27 (m, 1H), 4.73–4.81 (m, 1H), 4.88–5.00 (m, 1H), 6.62–6.69 (m, 1H), 6.95–7.01 (m, 1H), 7.17–7.46 (m, 7H); ^13^C-NMR (75 MHz, CDCl_3_): δ 24.1, 25.7, 27.1, 42.0, 43.5, 55.3, 76.3, 109.2, 122.8, 124.0, 125.1, 126.8, 127.5, 128.5, 128.6, 128.8, 129.6, 135.3, 143.4, 176.6, 211.3. Elemental analysis C_21_H_21_NO_3_: C 75.20, H 6.31, N 4.18. Found C 75.45, H 6.25, N 4.07. HPLC Chiralpak OD-H column, *n*-hexane/2-propanol = 95:05, flow rate 1.0 mL/min, λ = 254 nm; t_R_ 19.25 min (major) and 45.23 min (minor).

*(S)-1-Benzyl-3-hydroxy-3-(3-methoxy-2-oxopropyl)indolin-2-one* (**10d**). Yield 89%; ^1^H-NMR (300 MHz, CDCl_3_) δ 7.44–7.29 (m, 12H), 7.22 (td, *J* = 7.8, 1.3 Hz, 3H), 7.10–7.00 (m, 2H), 6.72 (d, *J* = 7.8 Hz, 2H), 4.92 (q, *J* = 15.7 Hz, 4H), 4.31 (s, 2H), 4.03 (s, 4H), 3.42 (s, 6H), 3.35 (d, *J* = 16.5 Hz, 3H), 3.03 (s, 2H). ^13^C-NMR (75 MHz, CDCl_3_) δ 40.1, 49.8, 50.9, 77.7, 78.3, 110.5, 123.0, 123.8, 126.9, 127.3, 128.5, 129.0, 129.8, 136.0, 144.5, 176.2, 210.0. Elemental analysis C_19_H_19_NO_4_: C 70.14, H 5.89, N 4.31. Found C 71.00, H 6.14, N 4.19. HPLC Chiralpak OD-H column, *n*-hexane/2-propanol = 90:10, flow rate 0.7 mL/min, λ = 254 nm; t_R_ 35.03 min (major) and 40.30 min (minor).

## 4. Conclusions

In conclusion, BINAM-prolinamides have shown to be efficient catalysts under solvent-free conditions, in terms of diastereo- and enantioselectivities, for the synthesis of non-protected and *N*-benzyl isatin derivatives. The achieved enantioselectivities are superior to those reported under solvent-free conditions using other prolinamide systems. However, these results are highly dependent on the structure of the isatin and the ketone used. The use of (*R*_a_)-BINAM-l-(bis)prolinamide as catalyst in the addition of cyclohexanone and α-methoxyacetone to free isatin led to 90% of *ee* and 97% *ee*, respectively. On the other hand, using acetone as nucleophile, good enantioselectivities were obtained with *N*-tosyl BINAM-l-prolinamide **4** as catalyst and *N*-benzyl isatin as electrophile with up to 90% *ee* being achieved. This good result has allowed the application of a silica-supported catalyst (**5**) that can be recovered and reused for at least five times without being detrimental on the results.
